# Distinct genetic programs drive antibiotic resistance and intracellular invasion in emerging MRSA strains

**DOI:** 10.1128/msystems.01396-25

**Published:** 2026-03-30

**Authors:** Sun Ju Kim, YuJin Shin, Seonmin Lee, Jihyeon Kim, Junggyeong Jang, Ji-Hoon Kim, Wonsik Lee

**Affiliations:** 1 School of Pharmacy, Sungkyunkwan University 65666, Suwon, Republic of Korea; Marquette University, Milwaukee, Wisconsin, USA

**Keywords:** *Staphylococcus aureus*, MRSA, ST72, USA300, antibiotic resistance

## Abstract

Methicillin-resistant *Staphylococcus aureus* (MRSA) is a major global health threat because of its ability to adapt. In North America, the USA300 lineage ST8 has become the predominant MRSA clone, whereas the ST72 lineage has emerged as an important MRSA in East Asia. Here, we compare USA300 and SAWL001 from the ST72 at the phenotypic, genomic, and transcriptomic levels. Phenotypic assays assessed antibiotic susceptibility, intracellular invasion, oxidative stress survival, biofilm formation under β-lactam exposure, blood-induced cell clumping, persister formation, and virulence in a mouse sepsis model. For genomic analyses, we compared the SAWL001 genome against USA300 and other major *S. aureus* strains. SAWL001 showed modestly higher resistance to rifampicin, gentamicin, and linezolid compared with USA300. We also found that SAWL001 *mecA* is inducible only under oxacillin, whereas USA300 *mecA* is constitutively expressed. Consistent with these differences, SAWL001 invaded human epithelial cells far less efficiently and survived H_2_O_2_ exposure at a significantly lower rate than USA300. Furthermore, our genome analysis revealed that SAWL001 has features different from USA300, such as the beta-lactamase gene locus. Finally, our transcriptomic profiling shows that USA300 maintains virulence features such as PVL, while SAWL001 shows adaptation toward greater horizontal gene transfer and antibiotic resistance. Together, our findings highlight that MRSA lineages can branch toward different evolutionary trajectories, such as becoming more antibiotic resistant or more invasive, underscoring the need for lineage-specific analysis to identify competence determinants and to tailor treatment strategies to each clone’s strengths and weaknesses.

**IMPORTANCE:**

Methicillin-resistant *Staphylococcus aureus* remains a leading cause of antibiotic-resistant infections worldwide, and its lineages can differ widely in antibiotic resistance and virulence. In this study, we compared the North American USA300 lineage (ST8) with an emerging East Asian ST72 strain, SAWL001. SAWL001 showed higher resistance to several antibiotics than USA300, although the overall resistance levels were moderate. Also, SAWL001 exhibits an inducible *mecA-*mediated methicillin resistance, whereas USA300 expresses *mecA* constitutively. Conversely, USA300 invades host epithelial cells more effectively and survives oxidative stress better than SAWL001. Genome and transcriptome analyses show that USA300 retains classical virulence factors, while SAWL001 is primed for horizontal gene acquisition. Our findings underscore distinct evolutionary strategies: USA300 appears to favor aggressive virulence, whereas SAWL001 shows greater metabolic and genomic flexibility, suggesting the need for lineage-specific control strategies.

## INTRODUCTION

*Staphylococcus aureus* continuously poses a critical public health challenge due to its remarkable capacity to develop resistance to antibiotics. Methicillin-resistant *S. aureus* (MRSA) infections are responsible for significant global morbidity and mortality, and it is estimated that the continuous increase in antimicrobial resistance (AMR) will lead to 8.2 million AMR-related deaths by 2050 (1). Limited development of new antibiotics for MRSA, together with its increasing incidence with age, makes it important to understand the emergence and dissemination of new MRSA clones (2–4). Since its first identification in the early 1960s, MRSA has evolved and disseminated worldwide, shaping regional variations of clonal dominance. Within the MRSA ST8 lineage, the USA300 strain emerged in the early 2000s as a major community-associated MRSA clone in North America, remarkable for its advantageous genetic make-up and high virulence (5). Notably, USA300 carries the SCC*mec* type IV and the Panton-Valentine leukocidin (PVL) genes, which have been attributed to its ability to cause severe skin and soft tissue infections and bacteremia, leading to USA300 becoming a major cause of MRSA infections (6).

Emergence of MRSA with non-dominant genotypes has been reported globally (7–10). This regional clonal replacement suggests that the evolution of MRSA lineages was driven by distinct selective pressures, such as antibiotic regimens and host factors. A notable example of MRSA clonal replacement is the predominance of ST72 in Korea over the last two decades and the recent dissemination in the East Asian countries (3, 11). These MRSA ST72 strains show genetic differences from USA300. For example, ST72 isolates encode an SCC*mec* type IV, as does USA300, but lack the PVL genes, suggesting that ST72 MRSA strains may rely on alternative virulence determinants such as staphylococcal enterotoxins to cause infection (12). Furthermore, ST72 MRSA strains are known to have a different antibiotic resistance profile. As ST72 MRSA strains have become prevalent, recent epidemiological studies have shown ST72 infections to be increasingly common in both community and healthcare facilities in South Korea, suggesting this lineage possesses competitive adaptability and has spread rapidly despite the lack of PVL (13, 14). The emergence of the ST72 MRSA clone in East Asia underscores the evolutionary adaptability of *S. aureus* and the need for continued surveillance of emerging variants. Given that current antibiotic regimens for MRSA treatment are largely based on clinical experience with USA300, a more comprehensive characterization and understanding of ST72 is a key step toward developing more effective strategies for preventing its further dissemination and improving treatment outcomes.

Here, using an ST72 MRSA isolate from Korea, named SAWL001, we performed a comprehensive and comparative analysis against the representative ST8 MRSA isolate, USA300. We examined differences in their antibiotic resistance profiles and key virulence-related phenotypes, generated the complete genome sequence of SAWL001 to identify its unique genetic features, and compared the global gene expression patterns between the two strains. We identified major genomic and transcriptomic differences underlying the distinct phenotypes of SAWL001 and USA300. By delineating these differences, our study provides new insights into the adaptation and dissemination strategies of MRSA clones, which can help to monitor and control emerging MRSA clones.

## RESULTS

### Differential *mecA* expression in SAWL001 and USA300 upon β-lactam exposure

Different MRSA clones have become predominant in different countries (15). In particular, ST72 has emerged as one of the predominant MRSA lineages in Asia, including South Korea. For this study, we used a clinically verified ST72 isolate, SAWL001, obtained from a blood specimen in a case of invasive infection. In a phylogenetic medoid tree analysis of 50 ST72 genomes, SAWL001 exhibited the lowest average pairwise distance (0.0127), suggesting that SAWL001 is representative of the ST72 lineage (Fig. S1).

The molecular type was determined by multilocus sequence typing (MLST) for the sequence type and by multiplex PCR for the staphylococcal cassette chromosome *mec* (SCC*mec*) type. For comparison, we selected five *S*. *aureus* reference strains, including three methicillin-sensitive *S. aureus* (MSSA) strains, HG003, Newman, and RN4220, and two MRSA strains, MW2 and USA300. For the MRSA strains, endogenous plasmid-cured derivatives were used (16, 17); therefore, plasmid-encoded antibiotic resistance determinants, including erythromycin resistance, were excluded.

**Fig 1:**
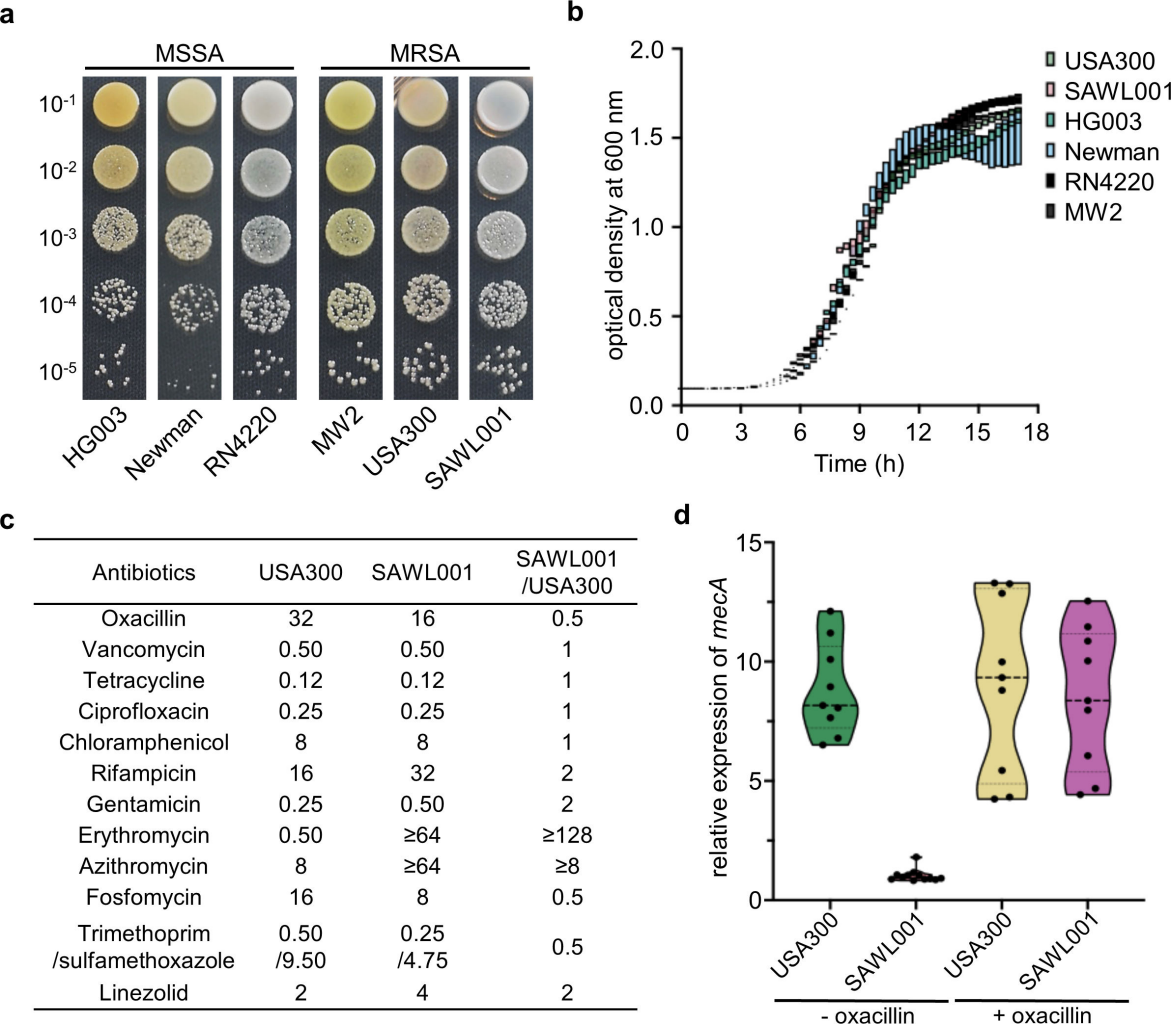
Microbiological characteristics of SAWL001 and type *S. aureus* strains. (**a**) CFUs of SAWL001 were compared with those of *S. aureus* reference strains, HG003, Newman, RN4220, MW2, and USA300, at the same optical density at 600 nm (OD_600_). (**b**) Growth curves of SAWL001 and the reference strains used in (**a**) were also compared in tryptic soy broth (TSB). (**c**) The minimum inhibitory concentrations of USA300 and SAWL001 were evaluated and compared as a ratio. All concentrations are given in μg/mL. Trimethoprim and sulfamethoxazole were tested in combination, with each compound’s concentration presented separately. (**d**) The relative expressions of *mecA* of USA300 and SAWL001 were compared with and without 1 µg/mL of oxacillin. Gene expression was normalized to *gapA* as the housekeeping gene. Data are represented as mean ± SD (*n* = 9).

First, the growth of SAWL001 was compared to that of the reference strains. All *S. aureus* strains showed no significant difference in growth rate, final optical density, and colony-forming units (CFUs) per OD at 600 nm (Fig. 1a and b). For subsequent analyses, the generally accepted reference ST8 strain, USA300, was used as the counterpart of SAWL001. We then measured the antibiotic susceptibility of both strains to representative antibiotics across different classes (Fig. 1c). Both strains were resistant to oxacillin, chloramphenicol, and rifampicin, but SAWL001 was resistant to macrolides (≥64 µg/mL). The minimum inhibitory concentration (MICs) for erythromycin and azithromycin in SAWL001 are due to a chromosomally encoded macrolide resistance gene. Because we used a plasmid-cured USA300 derivative, the parental USA300 strain, which retains the macrolide resistance plasmid, would be expected to exhibit erythromycin resistance comparable to SAWL001 (18). Nonetheless, SAWL001 carries the macrolide resistance gene on the chromosome, whereas USA300 carries it on a plasmid, which may result in reduced stability of macrolide resistance in USA300 due to plasmid loss. Indeed, approximately 5% of clinical USA300 isolates lack this plasmid (19).

We next examined the regulation of *mecA* expression under β-lactam exposure, since the genetic program of SCC*mec* is critical for the response to β-lactams. Interestingly, SAWL001 showed a lower final growth yield than USA300 when exposed to a sublethal dose of oxacillin (2.5 µg/mL) (Fig. S2), suggesting a strain difference under β-lactam treatment. We then measured *mecA* expression levels in SAWL001 and USA300 after 1 µg/mL oxacillin treatment for 30 min. Interestingly, SAWL001 showed a significant increase in *mecA* expression upon oxacillin exposure, whereas USA300 maintained a consistent level of *mecA* expression regardless of treatment (Fig. 1d). This *mecA* induction in SAWL001 was specific to β-lactam antibiotics, but not other classes (Fig. S3). Both SAWL001 and USA300 have SCC*mec* type IV, which contains *mecA* but lacks the canonical regulatory genes, *mecI* and *mecR1*, the repressor and the sensor inducer for *mecA*, respectively (20). Given this shared absence, a constitutive *mecA* expression in USA300 is expected. However, the inducible *mecA* expression in SAWL001 suggests involvement of other regulatory elements outside the SCC*mec* region.

### SAWL001 is less invasive than USA300 and more susceptible to oxidative stress

**Fig 2:**
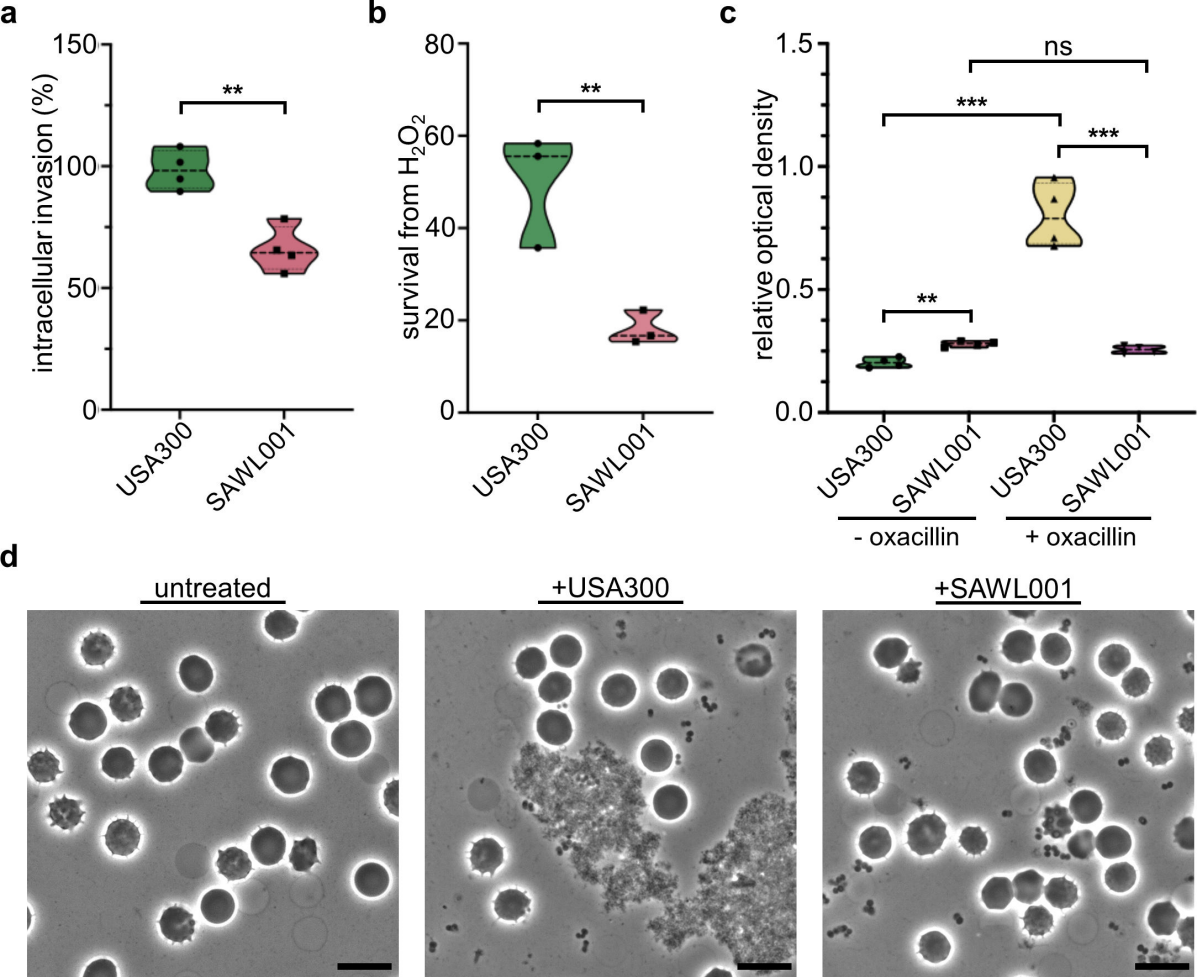
Comparison of SAWL001 and USA300 for infection-related characteristics. (**a**) Intracellular invasion of SAWL001 and USA300 into A549 cells was determined by cell infection assay (intracellular invasion [%] = recovered CFU/input CFU × 100). (**b**) The survival of SAWL001 and USA300 was compared after exposure to hydrogen peroxide for an hour (survival from H_2_O_2_ [%] = CFU after treatment/initial CFU × 100). (**c**) Biofilm formation of SAWL001 and USA300, with and without oxacillin treatment, was measured by the optical density of crystal violet staining at 550 nm. Biofilm formation was normalized based on growth (OD_600_). Data in panels **a–c** are presented as the mean ± SD (**a and c**, *n* = 4; **b**, *n* = 3 biological replicates), and the *P-*values are calculated by a two-sided, unpaired Student’s *t*-test. (**d**) Microscopic analysis of human whole blood treated with USA300 and SAWL001 for overnight. Compared with the untreated group, the treated groups show clumping. The scale bars represent 10 μm.

To further compare virulence-associated phenotypes of SAWL001 and USA300, we assessed key phenotypes relevant to intracellular or extracellular infection. For intracellular infection, we measured intracellular invasion efficiency and survival under oxidative stress. Compared with USA300, SAWL001 shows markedly reduced efficiency of intracellular invasion into human alveolar epithelial A549 cells, with an average entry efficiency of 65.83% for SAWL001 versus 98.55% for USA300 (Fig. 2a). Next, we compared the strains’ survival under hydrogen peroxide exposure to mimic the effects of the host’s innate immunity. SAWL001 was significantly more susceptible, surviving at an average rate of 18.09%, which was approximately one-third the survival rate of USA300 (49.87%) (Fig. 2b). These results suggest that SAWL001 is less capable of invading host cells and less tolerant of oxidative stress than USA300. This could make it more susceptible to innate immune responses during infection. Our findings align with previous reports showing that the presence of PVL and other aggressive virulence factors in USA300 contributes to high cytotoxicity and invasive capacity (21, 22). Conversely, the absence of PVL in the ST72 lineage, to which SAWL001 belongs, corresponds with reduced invasiveness.

For extracellular infection, we evaluated biofilm formation, clumping, and persister cell formation. In the absence of antibiotic pressure, SAWL001 formed more biofilm (an average OD_600_ 0.279) than USA300 (an average OD_600_ 0.203), whereas under oxacillin exposure, the biofilm produced by USA300 (an average OD_600_ 0.802) surpassed that of SAWL001 (an average OD_600_ 0.255) (Fig. 2c). In the clumping test, USA300 showed more cell clumping in liquid culture without oxacillin (Fig. 2d), suggesting stronger cell aggregation than SAWL001 under the same conditions. In the persister formation test, we did not observe a significant difference between SAWL001 and USA300 under high-dose gentamicin (100× MIC) treatment, as shown in Fig. S4. Furthermore, we measured the 50% lethal dose (LD_50_) in a murine infection model to assess whether this difference was consistent. As shown in Fig. S5, at 5 × 10⁸ CFU, the SAWL001-infected group displayed approximately 30% more survival, consistent with reduced virulence of SAWL001 compared with USA300. These observations suggest that USA300 would have a more aggressive infection profile, greater survival against host responses, and stronger adaptive responses to stress than the ST72 clone, SAWL001. Conversely, despite its successful spread in East Asia, SAWL001 may possess other fitness advantages, such as antibiotic resistance, at the expense of being comparatively less invasive.

### SAWL001 chromosome has a CC8 backbone, but more emphasis on metabolism

**Fig 3:**
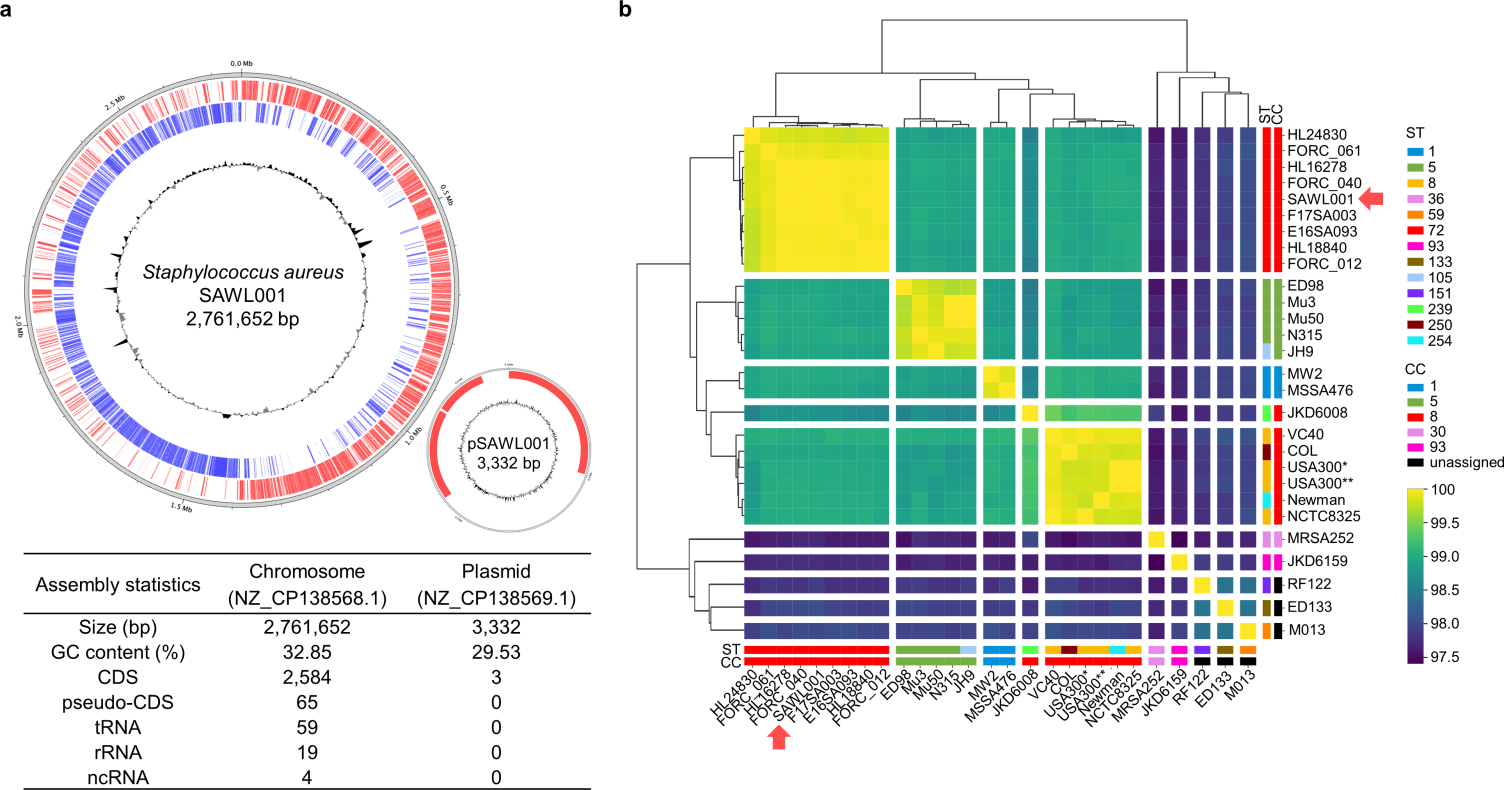
Whole-genome sequence of SAWL001 and ANI table. (**a**) Genome map of the SAWL001 chromosome and plasmid pSAWL001, which encodes three genes. (**b**) A phylogenetic tree was reconstructed based on ANI and sequence types (STs) and clonal complexes (CCs) of the type strains that have complete genome sequences available in KEGG are visualized as a heatmap. MLST defines isolates by STs, which are unique combinations of alleles from housekeeping genes. CCs are groups of closely related STs that share a recent common ancestor, typically differing at only one or a few loci. The CC8 clonal complex encompasses multiple sequence types, including ST59, ST72, ST239, ST150, and ST254, among which SAWL001 belongs to ST72. The columns and rows were separated by clades from the UPGMA-based phylogenetic tree. *: USA300_TCH1516; **: USA300_FPR3757.

The plasticity of *S. aureus* metabolism and virulence contributes to its global spread. These properties are partly linked to the remarkable clonal diversity of successful local MRSA clones, especially lineages in Asia (23, 24). To identify genomic features of one of the most successful clones, SAWL001, we sequenced its whole genome using both short- and long-read sequencing. After generating a complete genome assembly, we performed comparative genomic analyses against USA300 and other *S. aureus* strains. SAWL001 possesses a chromosome of 2,761,652 bp (NZ_CP138568.1) and a plasmid of 3,332 bp (NZ_CP138569.1). The chromosomal GC content is 32.85%, and the plasmid GC content is 29.53% (Fig. 3a). The chromosome encodes 2,584 predicted coding sequences, along with 65 pseudogenes, 59 tRNA genes, 19 rRNA genes, and 4 ncRNAs. The plasmid harbors three genes: *rep* encoding a replication protein, *cadD* encoding a cadmium resistance protein, and an *arsR/smtB* family transcription factor that functions as a metalloregulator. Our average nucleotide identity (ANI) analysis of representative *S. aureus* isolates revealed that ST72 isolates form a distinct clade, separate from the clade comprising ST1, ST5, and ST8 (Fig. 3b). These observations suggest that the phenotypic differences described above, including attenuated virulence, reflect differences in genetic background. Consistent with this, comparative genomic analysis of USA300 and SAWL001 revealed substantial chromosomal divergence (Fig. S6).

We next compared the functional classes of the annotated genes of SAWL001 and USA300. Genome annotation analysis suggested that, aside from hypothetical genes, the largest category of genes in SAWL001 is amino acid transport and metabolism (COG category E) (Fig. S7). This emphasis on metabolism may reflect enhanced metabolic capacity in SAWL001, potentially contributing to improved survival during antibiotic treatment in infection, despite largely similar core genomic content. Overall, our systematic genomic comparison suggests that SAWL001 retains the conserved backbone of CC8 lineages as in USA300, yet harbors genetic elements that may facilitate adaptation to antibiotic stress.

### SAWL001 has multiple antibiotic resistance determinants along with rapid inducible *mecA*

**Fig 4:**
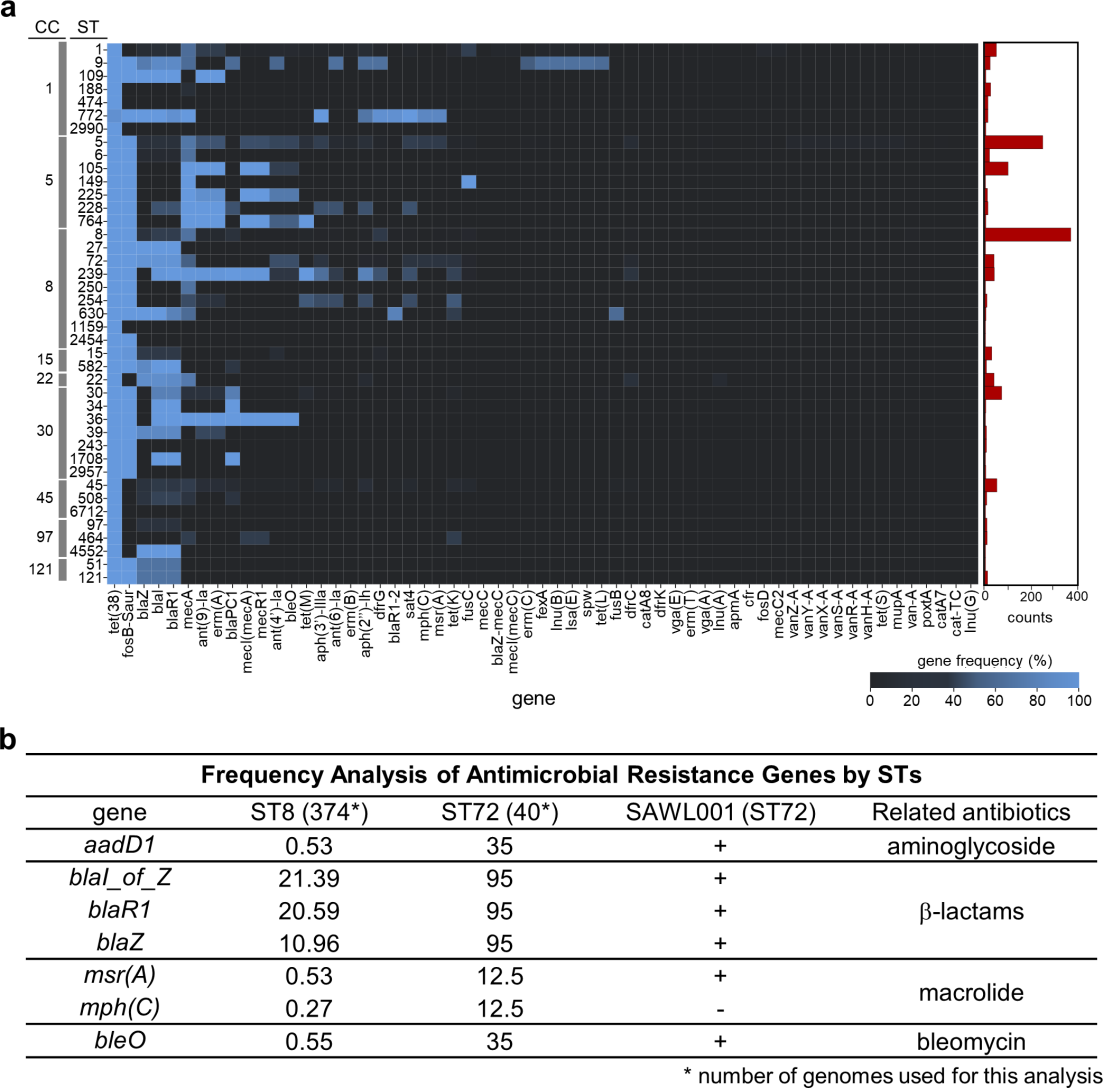
Population genomics of *S. aureus.* (**a**) The frequency of antibiotic resistance and virulence genes in each sequence type was identified and visualized as a heatmap. The number of samples in each ST is depicted as a bar graph on the right. Genes associated with tetracycline resistance were the most frequently observed, followed by genes conferring resistance to fosfomycin (*fosB*-Saur) and β-lactam antibiotics, which also showed high prevalence. The right panel represents the number of isolates in each sequence type. (**b**) The genes showing the greatest difference in frequency between ST8 (including USA300) and ST72 (including SAWL001) are compared. In ST72 strains, genes conferring resistance to aminoglycosides, β-lactams, macrolides, and bleomycin were observed at higher frequencies. Among these, SAWL001 lacks only the macrolide-associated *mph(C*) gene.

To investigate which genetic factors might underlie the success of ST72, we performed a population genomic analysis of 2,052 complete *S. aureus* genomes available in a public database. Particularly, we sought to identify antibiotic resistance and virulence genes associated with dominant clones such as ST72. We found that several resistance genes are present in nearly all *S. aureus* lineages. These include the tetracycline efflux pump (*tet38*), the fosfomycin resistance gene (*fosB*), and the beta-lactamase operon (*blaZ blaR1 blaI*) (Fig. 4a; Fig. S8). Comparing ST72 including SAWL001 and related isolates with ST8, including USA300 and its relatives, revealed seven genes that differ markedly in prevalence between the two lineages. These genes confer resistance to four classes of antibiotics: aminoglycosides, beta-lactams, macrolides, and bleomycin. Notably, 95% of ST72 isolates (38/40) carried the β-lactamase operon (*blaZ blaR1 blaI*), whereas less than 25% of ST8 isolates carried the locus. This enrichment of the β-lactamase regulatory locus in ST72 could provide a strong advantage where β-lactam antibiotics are used frequently, for example, in East Asia, which has relatively high antibiotic prescription rates (25, 26). Also, SAWL001 carries additional determinants of resistance to major antibiotic classes, as appears in other ST72 strains. These include resistance genes for aminoglycosides and glycopeptides, but not the macrolide phosphotransferase gene *mphC* (Fig. 4b). Therefore, SAWL001 represents the characteristic resistance profile of the ST72 lineage.

**Fig 5:**
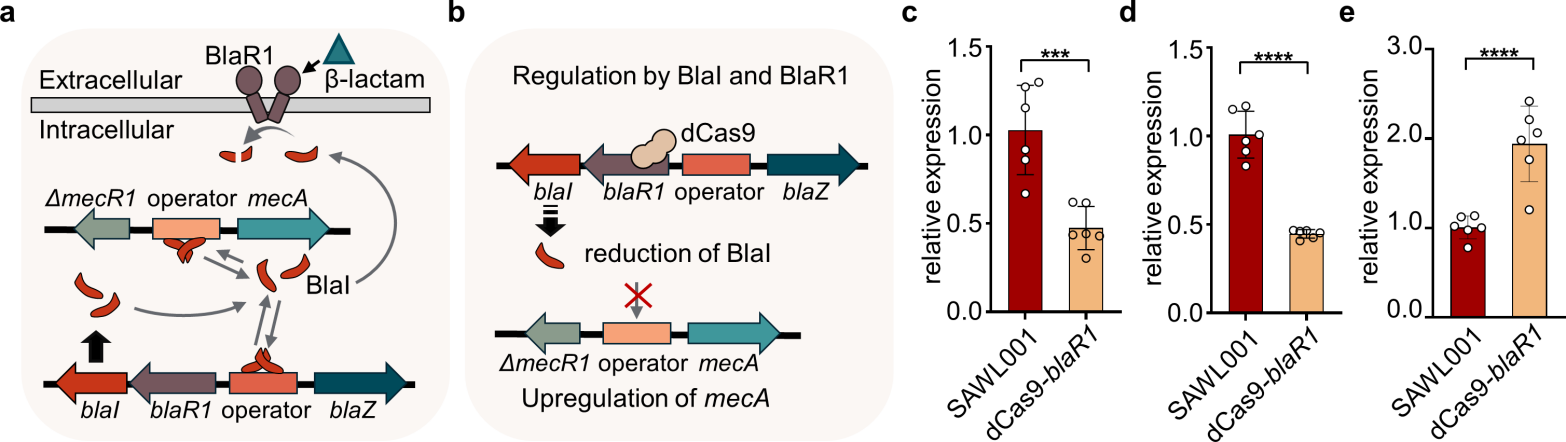
The inducibility of *mecA* expression by the *bla* system in SAWL001. (**a**) The schematic illustrates the regulation of *mecA* and *blaZ* by the *bla* system in SAWL001, featuring SCCmec type IV and *bla* operon. (**b**) The schematic shows the change in regulation of *mecA* expression by the interference of the *bla* system using CRISPRi. (**c–e**) The relative expressions of *blaR1* (**c**), *blaI* (**d**), and *mecA* (**e**) were compared between SAWL001 and *blaR1* silencing strain by CRISPRi. Data in panels **c–e** were normalized to *gapA* and are expressed as the relative levels to SAWL001. Data are represented as mean ± SD (*n* = 6), and the *P-*values are calculated by a two-sided, unpaired Student’s *t*-test.

Importantly, the presence of *blaR1* and *blaI* in SAWL001 aligns with its inducible *mecA* expression upon β-lactam exposure (Fig. 1d). The sensor transducer BlaR1 and the repressor BlaI correspond functionally to MecR1 and MecI, which are absent in SCC*mec* IV (27–29). Thus, BlaR1 BlaI can substitute for the missing MecR1 MecI and render *mecA* inducible in SAWL001, suggesting potential crosstalk between these systems during β-lactam-dependent *mecA* expression. This arrangement may also provide an advantage in its expression, since *bla*-mediated induction of *mecA* can occur within minutes of β-lactam exposure as noted, whereas the canonical *mec* system usually responds over several hours (30). Moreover, BlaI yields weaker repression of *mecA* than MecI, which is a tighter repressor (30), consistent with our results above that SAWL001 maintains a low basal level of *mecA* expression even without oxacillin (Fig. 1d). This mechanistic link between the *bla* system and *mecA* regulation illustrates how the *bla* system contributes to β-lactam resistance in MRSA SCCmec IV type clones (Fig. 5a). To validate this regulation, we performed a *blaR1* silencing assay using CRISPRi to interfere with the *bla* system and determine its effect on *mecA* expression levels in SAWL001 (Fig. 5b). As expected, *blaR1* silencing led to a substantial reduction in *bla* expression (Fig. 5c and d), consistent with the genetic context of the *bla* locus. This repression was accompanied by a marked induction of *mecA* expression in SAWL001, thereby validating our genomic analysis (Fig. 5e). Furthermore, our findings suggest that because the β-lactamase operon (*blaZ blaR1 blaI*) is present in most ST72 isolates, ST72 strains that carry *mecA* are likely to show a m*ecA* regulatory circuit similar to that in SAWL001.

### Transcriptomic analysis reveals the distinct difference in virulence and abundance of antibiotic resistance genes between USA300 and SAWL001

**Fig 6:**
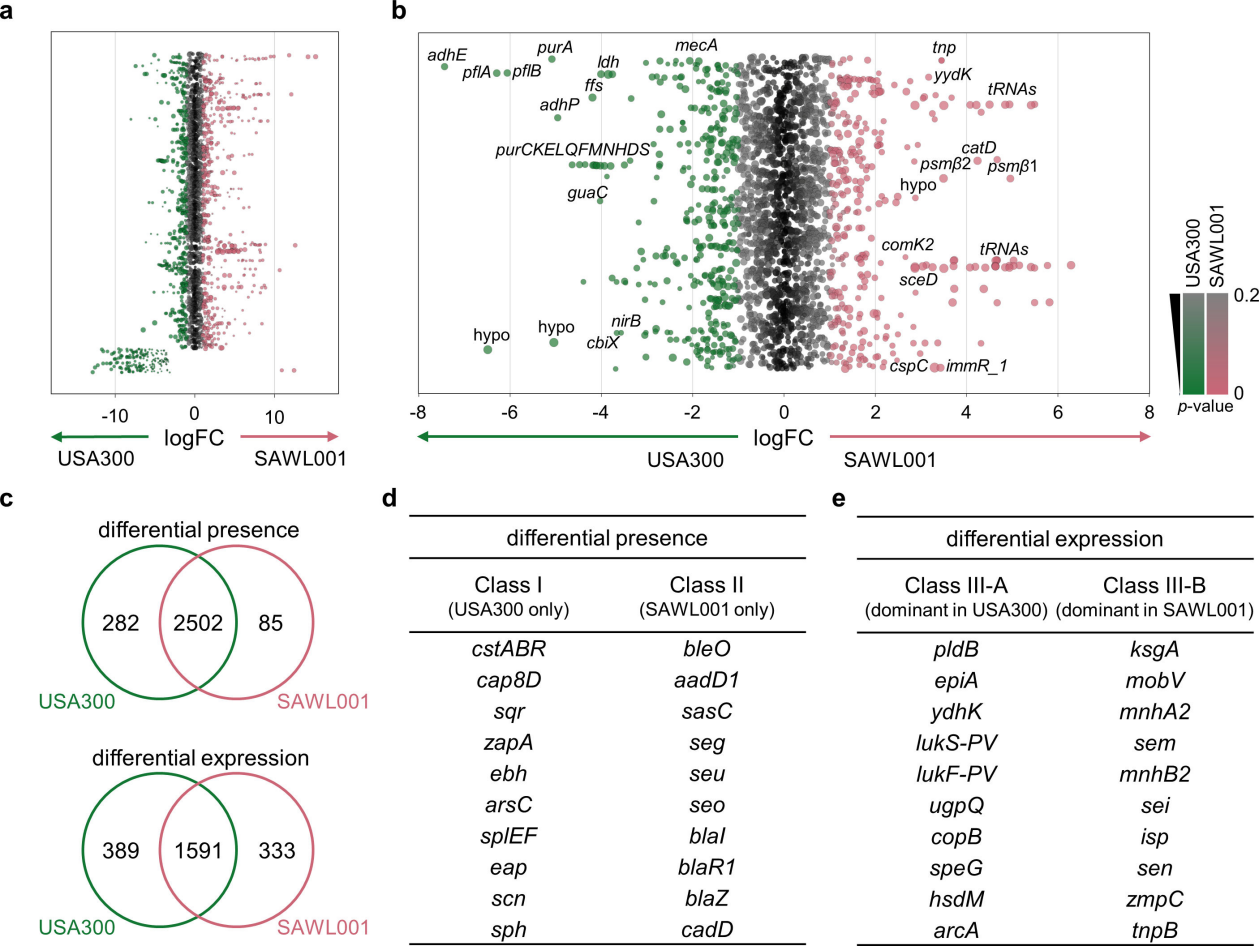
Transcriptome comparison between SAWL001 and USA300. (**a**) The fold changes of genes in log base 2 scale are depicted as dots. Dot size is related to the CPM. The genes are aligned by the order in database annotation. Genes outside of the red box correspond to those showing differential presence between the strains. (**b**) The fold changes of core genes in log base 2 scale are depicted as dots. The size of the dot is also related to the CPM, in the same scale as in panel **a**). *P*-values are represented using two colors—green for USA300 and pink for SAWL001—with color intensity corresponding to *P*-value. (**c**) The Venn diagram shows genes with differential presence and differential expression in USA300 and SAWL001. Both strains share 2,502 genes with identical presence and 1,591 genes with similar expression levels. However, 282 genes are unique to USA300 and 85 to SAWL001, respectively, and 389 and 333 genes showed higher expression in USA300 and SAWL001, respectively. (**d and e**) Ten representative genes, with known gene symbols, are listed for each strain in the categories of differential presence and differential expression.

Although analysis of genomic features and genes that determine phenotype provides important insight into this strain, gene expression profiling often elucidates the phenotype in more detail. Therefore, in addition to the genomic comparison above, we performed a comparative transcriptomic analysis using RNA-seq to identify differential gene expression profiles between USA300 and SAWL001. For this analysis, both strains were cultured in tryptic soy broth (TSB) medium and sampled at the exponential phase. Although the two strains are closely related and share the highest ANI among the tested strains, both contain sets of unique genes. To enable a fair comparison, we constructed a combined reference genome, a pan-genome for the two strains for RNA-seq, by merging the transcriptomes of both strains. This approach allowed us to identify and quantify RNA transcripts present in one strain but not the other. The pan-genome included 2,869 transcripts (Fig. 6). Global gene expression profiles showed that each strain had a distinct transcriptional pattern, with numerous genes significantly upregulated in one strain relative to the other (Fig. 6a and b). In particular, *mecA* was expressed at higher levels in USA300 than in SAWL001, consistent with Fig. 1d. Of the 2,313 core genes shared by both strains, 1,591 (68.8%) showed no significant difference in expression, whereas 389 and 333 genes were more expressed in USA300 and SAWL001, respectively (|fold change| > 2, *P* < 0.05). Additionally, 282 transcripts were detected exclusively in USA300 and 85 exclusively in SAWL001, reflecting strain-specific gene content (Fig. 6c). Interestingly, SAWL001 expresses a higher proportion of genes belonging to COG category C (energy production and conversion), E (amino acid transport and metabolism), and P (inorganic ion transport and metabolism) than USA300 (Fig. S9).

Among differentially expressed genes (Fig. 6d), USA300 appears to express genes encoding proteases such as *splE*, *splF*, and *sph* that are absent in SAWL001, whereas SAWL001 exclusively expressed genes encoding enterotoxins such as *seg*, *seo*, and *seu*, and *aadD1* encoding an aminoglycoside-modifying enzyme that is not found in USA300. Although SAWL001 expresses those enterotoxin genes, these factors are known to be less potent in acute virulence than PVL or other major toxins found in USA300. We further found that SAWL001 showed higher expression of *comK2*, which is involved in genetic competence and gene expression regulation, even during the exponential growth phase when competence genes are usually repressed (31). Notably, elevated *comK2* levels can indirectly repress certain virulence genes (31). For example, overexpression of competence regulators has been reported to inhibit the expression of adhesins and exotoxins, including clumping factors encoded by *clfA* and *clfB*, as well as the two-component system *saeRS* controlling many virulence factors. Therefore, its elevated expression in SAWL001, along with potential upstream activators like *sigH* and *comK1*, is consistent with SAWL001’s lower clumping ability and reduced invasiveness observed above (Fig. 2). We also found *hsdM* to be highly expressed only in USA300 (Fig. 6e). This gene encodes a DNA methyltransferase component involved in restricting foreign DNA (32). This finding, together with SAWL001’s enhanced competence and transposase activity, suggests that USA300 invests in preventing horizontal gene transfer, whereas SAWL001 may be more permissive. Additionally, SAWL001 showed elevated expression of a transposase gene associated with a mobile genetic element carrying multiple resistance genes, which may contribute to the dissemination of resistance determinants within the clone. Furthermore, in USA300, genes in the *de novo* purine biosynthesis pathway, including *purA, purK, purE, purL, purQ, purF, purM, purN, purH, purD, purS*, and *guaC*, were expressed at higher levels than in SAWL001. Upregulation of purine metabolism has been implicated in enhanced virulence in *S. aureus*, such as increased intracellular invasion and biofilm formation, due to increased availability of purine nucleotides and related metabolites (16, 33–35). Furthermore, we found that the elevated expression of *agr* genes (*agrA*, *agrC*, and *agrB*) and *sspA*, which encodes the V8 protease, in USA300 compared with SAWL001 may contribute to increased virulence, in addition to the presence of PVL in USA300. These findings are consistent with our previous results (Fig. 2), which show that SAWL001 is less efficient in intracellular invasion and more susceptible to oxidative stress. Our integrated analyses reveal that SAWL001 has adopted a distinct survival strategy, providing mechanistic context for the phenotypic features to SAWL001. This suggests that SAWL001 has become less invasive but more flexible in its metabolism.

## DISCUSSION

Our data reveal that SAWL001 has adopted a resistance-first strategy, trading off virulence for extensive drug resistance. SAWL001 encodes additional resistance loci, for example, the β-lactamase operon, that USA300 lacks. Although both SAWL001 and USA300 have SCC*mec* IV, SAWL001 uniquely induces *mecA* under β-lactam antibiotic exposure. In contrast, USA300, which lacks a functional *blaR1-blaI* system, maintains *mecA* expression, which may enable a more rapid response to β-lactam antibiotics, including oxacillin, during antibiotic treatment in the course of infection. Additionally, the multiple antibiotic resistance observed in SAWL001, although moderate, may reflect intrinsic resistance factors, including chromosomally encoded antibiotic efflux pumps, whereas USA300 seems to possess a more streamlined genome optimized for pathogenicity. This difference is consistent with the higher mortality observed in USA300-associated sepsis compared with ST72-associated sepsis (USA300: 29.8%, 20 of 67 cases; ST72: 16.5%, 13 of 79 cases) (36, 37).

Consistent with these genetic differences, SAWL001 was significantly less virulent and less stress tolerant than USA300. SAWL001 lacks the PVL phage and other USA300-specific virulence islands, relying instead on core toxins. Previous work showed that in PVL-negative ST72, Agr-regulated factors such as PSMs dominate neutrophil and erythrocyte lysis (38). The phenotypes of reduced neutrophil killing, decreased invasion, and lowered hydrogen peroxide survival align with an overall reduction in toxin production. By contrast, USA300 combines PVL, ACME, and a hyperactive Agr to achieve hypervirulence, contributing to its aggressive profile with high invasiveness and robust stress defenses (21, 22, 39). MRSA clones that carry large resistance elements often attenuate toxin production to offset fitness costs (40, 41). ST72 appears to have turned down virulence, allocating resources to resistance and biofilm, as we observed SAWL001’s higher biofilm than USA300.

Our transcriptomic analysis further demonstrates divergent strategies in SAWL001. USA300 upregulated *de novo* purine biosynthesis genes, which are wired to virulence regulation. PurR links purine metabolism to virulence, and loss of PurR makes *S. aureus* hypervirulent (34). Thus, USA300’s enhanced purine pathway likely fuels rapid growth and intracellular survival and may indirectly prime toxin expression by relieving PurR-mediated repression. In contrast, SAWL001 upregulated the competence regulator *comK2*. ComK2 is a late competence regulator whose regulon overlaps with genes controlled by SigH and ComK1 (31). SAWL001 also downregulated restriction systems, potentially reprogramming the cell to be more permissive. These features suggest that USA300 preferentially channels resources into a metabolic virulence program, whereas SAWL001 emphasizes genome plasticity and mobile elements that may facilitate acquisition of resistance factors. These contrasting strategies highlight how *S. aureus* can evolve along virulence-first or resistance-first trajectories, shaped by selective pressures in different host environments during infection.

Our comparative analysis of the USA300 and SAWL001 MRSA strains suggests that these clones may succeed by diverging along metabolic flexibility and virulence axes. SAWL001, an Asian ST72 clone, has accumulated genetic and metabolic flexibility, including intrinsic resistance factors, but exhibits weaker virulence, with reduced cell invasion and stress tolerance. By contrast, USA300 maintains strong virulence features, including potent toxins and active purine metabolism. These differences mirror clear genetic and transcriptomic distinctions. SAWL001 lacks PVL and favors genes for genetic uptake and adaptation, whereas USA300 emphasizes aggressive toxins and defense against foreign DNA. In conclusion, MRSA epidemiological success can arise through alternate paths, with USA300 strengthening virulence and fitness and ST72 emphasizing the flexibility of the chromosome. These findings emphasize the importance of tailored surveillance and control measures that account for clone-specific strengths and weaknesses. Understanding these trade-offs will aid in the prediction of emerging MRSA trends and inform targeted interventions.

## MATERIALS AND METHODS

### Bacteria and chemical compounds

The Korean clinical MRSA isolate SAWL001 is deposited in both the National Culture Collection for Pathogens (NCCP) of Korea as NCCP 14750 and the Leibniz Institute DSMZ (DSM) as DSM 110682 (42). It was isolated from a blood specimen in Changwon Fatima Hospital on 4th January 2005. The isolate was reported to be resistant to penicillin G, cefoxitin, and erythromycin, but susceptible to clindamycin, gentamicin, linezolid, vancomycin, ciprofloxacin, and tetracycline. SAWL001 is classified as ST72 and has SCC*mec* type IVa. The USA300 TCH1516 derivative generated by curing the endogenous plasmid pUSA300HOUMR was used in this study (16, 17). All bacterial cultures were incubated at 30°C in tryptic soy broth (TSB; Beckton Dickinson) with shaking at 250 rpm, or on tryptic soy agar (TSA; Beckton Dickinson), overnight unless otherwise noted. All antimicrobial agents used in this study were the products of Sigma-Aldrich (Burlington, MA, USA). All bacterial strains used in this study are summarized in Table S1.

### Phylogenetic analysis and estimation of pairwise genetic distances

To identify the most representative ST72 *Staphylococcus aureus* strain, all publicly available *S. aureus* complete genome assemblies were retrieved from NCBI GenBank (*n* = 2,564), and MLST was performed using the *S. aureus* MLST scheme to identify ST72 strains, yielding 52 genomes. A maximum-likelihood phylogenetic tree was constructed using IQ-TREE v2.3.6 with the GTR + G substitution model and 1,000 ultrafast bootstrap replicates, with USA300-FPR3757 (ST8) designated as the outgroup (43). For each ST72 strain, the average pairwise phylogenetic distance to all other ST72 strains within the tree was calculated. Pairwise distances, defined as the sum of branch lengths between two terminal nodes, were extracted using BioPython v1.83 (44). The strain exhibiting the lowest average pairwise distance was considered the most representative, as this metric reflects the most central phylogenetic position within the ST72 lineage. The country of origin for each strain was obtained from the geographic location metadata associated with the corresponding BioSample records.

### Antibiotic susceptibility testing

Antibiotic susceptibility testing (AST) was performed following the ISO 20766-2:2021 and CLSI guideline (45, 46). The interpretive criteria for susceptibility follow CLSI M-100 (Ed34), and EUCAST Clinical breakpoints (v 14.0) were used when CLSI M-100 was not available (47).

### Preparation for genomic analysis and qRT-PCR

For whole-genome sequencing, DNA was extracted from an overnight culture. The centrifuged bacterial cell pellet was treated with P1 buffer (Qiagen, Hilden, Germany) containing 300 μg/mL lysostaphin (AMBI, Lawrence, NY, USA) at 37°C, and GES buffer was added at RT. Then, protein in the samples was removed using chloroform after treatment with cold 7.5M NH_4_Ac. DNA precipitated by isopropanol was cleaned using ethanol and was eluted in 50% EB (Qiagen, Hilden, Germany).

To quantify bacterial RNA from USA300 and SAWL001, total RNA was isolated using the RNeasy kit (Qiagen, Hilden, Germany) at the log phase (OD_600_ ~0.5) according to the manufacturer’s instructions. The synthesis of cDNA and RT-PCR was carried out using RNA to cDNA EcoDry P remix (Takara, San Jose, CA, USA) and EzAMP qPCR 2× Master Mix with SYBR Green (Elpis-bioteck, Daejeon, Republic of Korea) according to the manufacturer’s instructions in consecutive order. Two hundred nanograms of RNA of each sample was equally added to 20 µL of reaction mixture. The RT-PCR was performed using LightCycler 480 instrument (Roche, Basel, Switzerland) with the following thermal program: 95°C for 10 min, and 40 cycles of 95°C for 15 s, 60°C for 15 s, and 72°C for 30 s. The primer sequences of target genes and internal control (*gapA*) are described in Table S2.

### *blaR1* silencing assay using CRISPRi

We performed the blaR1 repression experiment using CRISPRi as described previously (48). Briefly, guide RNAs (blaR1_sgRNA_Fw and blaR1_sgRNA_Rv) were cloned into plasmid pFD116 (#124769; Addgene, Watertown, MA, USA) containing dCas9 in *Escherichia coli* DH5α (17) and confirmed using primers blaR1_sgRNA_F and pFD116_sgRNA_R. The generated pFD116-*blaR1* plasmid then was transformed into RN4220. Transformants were recovered on TSB agar medium containing 200 µg/mL streptomycin. The resulting pFD116-*blaR1* was then transduced into SAWL001. For the *blaR1* silencing assay, SAWL001 was treated with 0.5 µg/mL Atet from the beginning of culture and cultured until OD_600_ reaches 0.4 as described (48). Total RNA was then extracted, and *blaR1*, *blaI*, and *mecA* transcript levels were quantified using the primers *blaR1_Fw*, *blaR1_Rv*, *blaI_Fw*, and *blaI_Rv* as described above. Guide RNA and primer sequences are provided in Table S2.

### Intracellular invasion rate

The experiment was designed based on gentamicin protection assay with minor modifications (49). The 2.5 × 10^5^ A549 cells in 0.5 mL RPMI 1640 (Welgene, Gyeongsan, Republic of Korea) containing 10% fetal bovine serum (FBS) (Welgene, Gyeongsan, Republic of Korea) were seeded in each well of a 24-well plate (SPL, Pochen, Republic of Korea) and incubated at 37˚C in 5% CO_2_ for 24 h for attachment. Overnight culture of each strain was inoculated in TSB media to make an OD_600_ 0.01 and incubated for 4 h until it reached the exponential growth phase. The bacterial cells were washed with sterile phosphate-buffered saline (PBS) twice and resuspended with new RPMI/10% FBS. For the invasion assay, RPMI/10% FBS with seeded A549 cells in 24-well plates was removed and replaced with 0.5 mL RPMI/10% FBS containing approximately 5 × 10^6^ CFU/mL bacteria at the multiplicity of infection (MOI) 10. The infected cells were incubated at 37°C in 5% CO_2_ for 1.5 h for invasion. RPMI with bacteria was removed, and cells were washed with PBS three times. It was replaced with RPMI with 200 µg/mL gentamicin, incubated for 1 h, and cells were washed three times with PBS to remove remaining gentamicin. The host cells were lysed with lysis buffer, 0.1% Triton X-100 (Sigma-Aldrich, Burlington, MA, USA) in PBS, and the invasive bacterial cells were collected with trypsin-EDTA solution (Welgene, Gyeongsan, Republic of Korea) thoroughly. The collected samples were serially diluted in PBS and spread on a TSA plate. The experiment was repeated four times.

### Hydrogen peroxide killing assay

The seed culture of USA300 and SAWL001 was inoculated into TSB media to an OD_600_ 0.05 and incubated until the culture reached OD_600_ 1.0. The 1 mL of culture was washed with 1 mL of PBS three times. The washed culture was diluted to make the OD_600_ 0.2 by PBS. The prepared culture was challenged with 40 mM hydrogen peroxide at 30°C for 1 h. The sampled culture was serially diluted in PBS at a 1:10 ratio, and each 5 µL of diluted samples was spotted on a TSA plate. The experiment was repeated three times.

### Biofilm formation assay

Biofilm formation assay was performed based on the method of O'Toole with minor modification (50). Briefly, the 4 × 10^6^ CFUs of overnight culture were inoculated 1:100 into fresh media, and 200 µL of the media was poured in a 96-well plate (SPL, Pochen, Republic of Korea). The plate was incubated overnight at 37°C for biofilm formation. The culture media was removed and fixed with 100 µL of methanol (Duksan, Seoul, Republic of Korea). The fixed biofilm was stained with 100 µL of 0.1% crystal violet (Sigma-Aldrich, Burlington, MA, USA) in water for 1 h. The remaining solution was removed, and the biofilm was washed three times with PBS. The remaining media was thoroughly removed and dried under hood for 10 min. The crystal violet was solubilized in 200 µL of 30% acetic acid (J. T. Baker, Phillipsburg, NJ, USA) at room temperature with agitation. The solubilized crystal violet was transferred to a new plate and serially diluted into a half, and the optical density at 550 nm was measured.

### Microscopic imaging for clumping assay

Overnight cultures of SAWL001 and USA300 were inoculated to an OD_600_ 0.01 in Single Donor Human Whole Blood Na Heparin (Innovative Research, IWB1NAH10ML) and incubated overnight at 37°C with agitation at 150 rpm. The 5 μL of sample from overnight blood culture was loaded on slide glass, and the coverslip was mounted on the sample. The slide glass was placed on the microscope (DMi8, Leica), and the image was acquired under the magnification of 1,000×.

### Bacterial persister assay

For persister assay, SAWL001 and USA300 were cultured in 25 mL TSB at 37°C with shaking until exponential phase (OD_600_ 0.5) and stationary phase (overnight), respectively. The bacterial pellets were collected by centrifuge and resuspended in PBS (OD_600_ 0.3) after being washed three times with PBS. To remove viable cells, the resuspended samples were treated with gentamicin at a final concentration of 100 μg/mL for 4 h, and the persister formation was compared with gentamicin-untreated condition by spotting the serial dilutions of each sample on TSA.

### Mouse systemic infection model

Specific pathogen-free (SPF) female, 7-week-old BALB/c mice were obtained from Orient Bio (Seongnam, Republic of Korea). The mice were acclimated for at least 1 week prior to infection and maintained under standard conditions with water and food *ad libitum*. Two strains, USA300 and SAWL001, were cultured in TSB at 30°C with shaking until they reached the exponential phase (OD_600_ 0.5). The bacterial pellets were harvested by centrifugation, washed twice with PBS, and resuspended in PBS to the desired concentrations. For the lethality assay, mice (*n* = 6 per group) were infected intraperitoneally with varying inocula of bacterial suspensions ranging from 1 × 10^7^ to 6 × 10^8^ CFUs. The exact infection dose was confirmed by plating serial dilutions of the inoculum on TSA plates. Survival was monitored for 48 h post-infection.

### Genome assembly

The short- and long-read sequences were produced by Illumina NovaSeq and PacBio Sequel, respectively. The initial sequences went through quality profiling and trimming by using fastp (v0.23.4) and LongQC (v1.2.0c) with default parameters. The complete genome sequence was assembled by using canu (v2.2) based on the hybrid assembly of short- and long-read sequences.

### Functional annotation and gene ontology assignment

To functionally characterize the predicted proteome, protein domains and families were assigned using InterProScan to search the comprehensive InterPro database, which integrates signatures from multiple member databases, including PANTHER. For the assignment of Gene Ontology (GO) terms, the predicted protein sequences were processed with eggNOG-mapper v2.1.12 against the eggNOG v5.0 database of orthologous groups (51). This analysis provided functional annotations, categorizing gene products based on their associated biological processes, molecular functions, and cellular components.

### *In silico* genotyping

*In silico* MLST was performed on the assembled whole-genome sequence to determine the isolate’s sequence type (ST). Alleles for the seven standard housekeeping loci for *S. aureus* were extracted and compared against the allelic profiles available in the PubMLST database to assign the corresponding ST.

### Population genomics of antimicrobial resistance genes and virulence factors

To investigate the distribution of AMR genes and virulence factors at the population level, a comprehensive genomic analysis was performed on a curated data set. A total of 2,052 complete *S. aureus* chromosome sequences were collected from the NCBI RefSeq database. Each genome was systematically screened to detect AMR and virulence-associated genes using a homology-based approach with ABRicate (v1.0.1). The screening was conducted against a custom-curated reference database composed of sequences from the Virulence Factor Database (VFDB) and the NCBI National Database of Antibiotic-resistant Organisms (NDARO). A gene was considered present in a genome if the alignment to a reference sequence met stringent thresholds of >90% identity and >80% coverage. The results were compiled into a percent presence matrix, grouped by the clonal complexes and sequence types.

### Genome visualization

A circular map of the SAWL001 genome was generated to visualize the arrangement of genomic features. The map was constructed using the Python package pyCirclize (v1.0.0). The key features of a genome sequence, including the positions of coding sequences on the forward and reverse strands, GC content, GC skew, and the locations of identified antimicrobial resistance genes and virulence factors, were denoted on the map.

### Whole-genome comparison

The overall genomic similarity between the isolate and other reference genomes was quantified by calculating the pairwise ANI computed using FastANI (v1.33) (52). To visualize the phylogenetic relationship among the strains, a distance matrix derived from the ANI values was used to construct a cladogram. The tree was generated using the unweighted pair group method with arithmetic mean (UPGMA) clustering algorithm (53).

### Gene orthology and comparative transcriptome analysis

For transcriptome profiling, SAWL001 and USA300 were grown in TSB medium at 30°C to the mid-exponential stage (OD_600_ 0.5). Total RNA was isolated from *S. aureus* cultures using the Qiagen RNeasy kit. Library preparation and RNA sequencing were conducted by Macrogen (Seoul, Republic of Korea). In brief, purified RNA was used to generate strand-specific cDNA libraries with the Illumina TruSeq mRNA sample preparation kit. The libraries were then sequenced using a paired-end Illumina platform and processed as described previously (54).

To establish high-confidence orthologous relationships between SAWL001 and the reference genome USA300_FPR3757, a consensus approach integrating two independent methods was employed. Orthologs were defined as gene pairs that satisfied criteria from both reciprocal best hit (RBH) analysis and orthologous group clustering. Briefly, an all-vs-all comparison of the CDSs from both genomes was conducted to identify RBHs using MMseqs2 easy-rbh (release 15-6f452). Following an initial search, any remaining, unmatched sequences were iteratively compared to find significant matches. A gene pair was accepted as an RBH if the match exceeded a simplified cosine similarity score of 0.4. The score was calculated as (alnlen × fident)/(qlen × tlen), where alnlen represents the alignment length, fident is the fractional identity, and qlen and tlen are the respective lengths of the query and target sequences. Concurrently, the protein sequences of both strains were merged and clustered into orthologous groups by MMseqs2 easy-cluster (release 15-6f452) (55). The final set of orthologs was determined by integrating the results of these two analyses (Fig. S10 and Fig. S11).

## Data Availability

All source code and raw data used for analyses and figures have been deposited on GithubGitHub (https://github.com/WonsikLeeLab/mSystems_SAWL001). The genomic and transcriptomic data were deposited in the NCBI database and are publicly available in BioProject (https://www.ncbi.nlm.nih.gov/sra/PRJNA1039545; BioProject accession number: PRJNA1039545). The whole-genome sequence of SAWL001 was uploaded on NCBI RefSeq and opened to the public under accession number GCF_040047685.1 (NZ_CP138568.1 for the chromosome and NZ_CP138569.1 for the plasmid pSAWL001).

## SUPPLEMENTAL MATERIAL

Supplemental material msystems.01396-25-s0001.pdf Fig. S1-S11; Tables S1 and S2.

ASM does not own the copyrights to Supplemental Material that may be linked to, or accessed through, an article. The authors have granted ASM a non-exclusive, world-wide license to publish the Supplemental Material files. Please contact the corresponding author directly for reuse.
