# Supplementary material for: Distinct genetic programs drive antibiotic resistance and intracellular invasion in emerging MRSA strains
Source: mSystems. 2026 Mar 30;11(4):e01396-25. doi: 10.1128/msystems.01396-25 (PMC13098256; doi:10.1128/msystems.01396-25)
Supplement: Supplemental material — Fig. S1-S11; Tables S1 and S2. [file msystems.01396-25-s0001.pdf]

Supplementary Data

Distinct genetic programs drive antibiotic resistance and intracellular invasion in emerging MRSA strains

Sun Ju Kim[1, †], YuJin Shin[1, †], Seonmin Lee[1], Jihyeon Kim[1], Junggyeong Jang[1], Ji-Hoon Kim[1], and Wonsik Lee[1,*]

[1] School of Pharmacy, Sungkyunkwan University, Suwon 16419, Republic of Korea

[†] Sun Ju Kim, YuJin Shin contributed equally to this work. Author order was determined alphabetically.

*Correspondence to: Wonsik Lee (wonsik.lee@skku.edu);

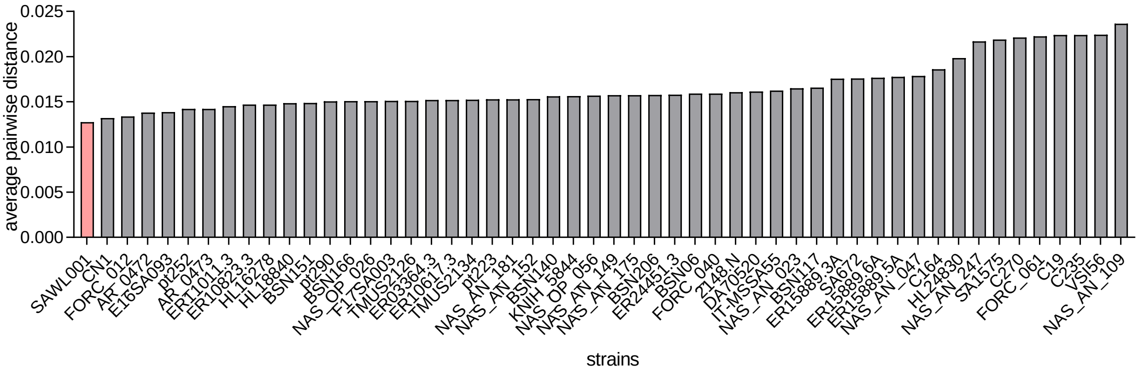

Fig. S1. Identification of the medoid of ST72 *Staphylococcus aureus*. Pairwise genetic distances were calculated from core single nucleotide polymorphisms (SNPs) across 52 complete chromosome sequences (51 publicly available and SAWL001). Analysis was performed using the GTR+G substitution model with 1,000 ultrafast bootstrap replicates (1-3). USA300-FPR3757 served as the outgroup.

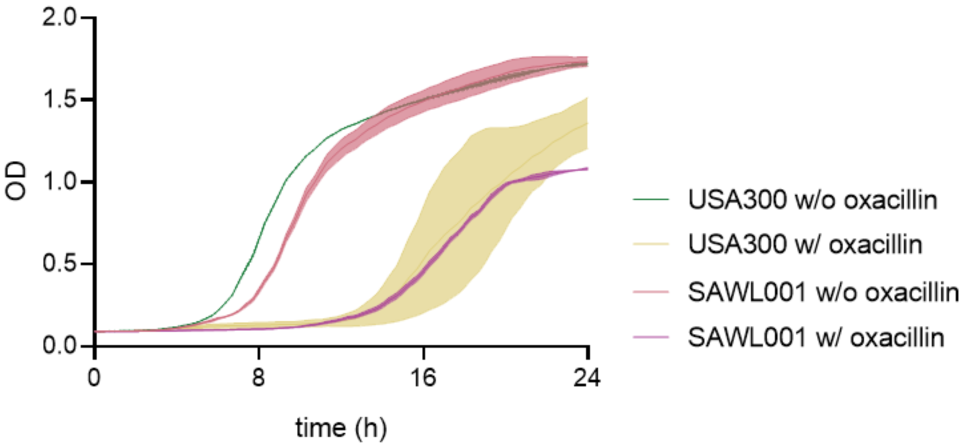

Fig. S2. Growth curve of USA300 and SAWL001 in the presence and absence of oxacillin. The concentration of oxacillin was used 2.5 μg/mL as sub-MIC of two MRSA strains.

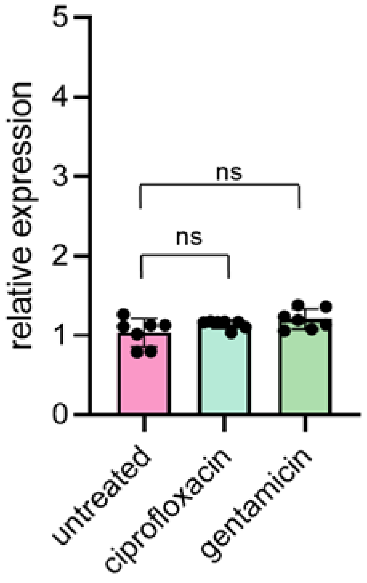

Fig. S3. The relative *mecA* expression of SAWL001 under non β-lactam antibiotic exposure. The relative expressions of *mecA* were measured after treating 0.016 μg/mL of ciprofloxacin and 0.032 μg/mL of gentamicin, respectively. The data were normalized to *gapA* as housekeeping gene and are expressed as fold change related to untreated expression level. Data are represented as mean ± SD (n = 7), and the *p*-values are calculated by a two-sided, unpaired Student's t-test.

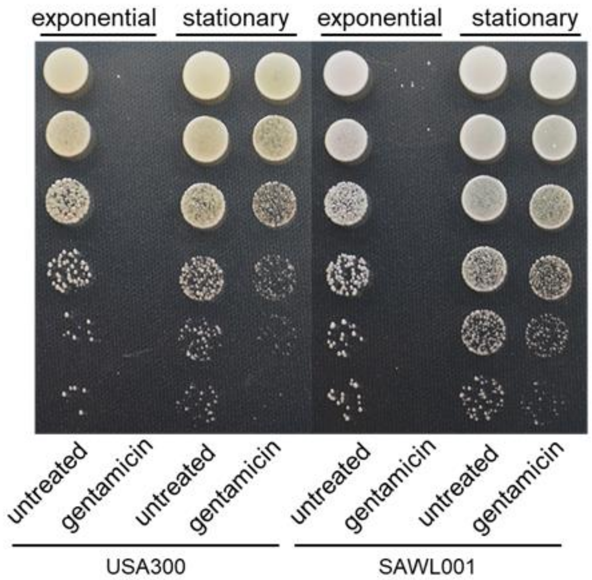

Fig. S4. The persister formation of USA300 and SAWL001 in the exponential and stationary phase. The persister cells was observed in gentamicin line, while Total bacterial cells in each phase was observed in untreated line. Treatment of gentamicin removed viable cells except persister.

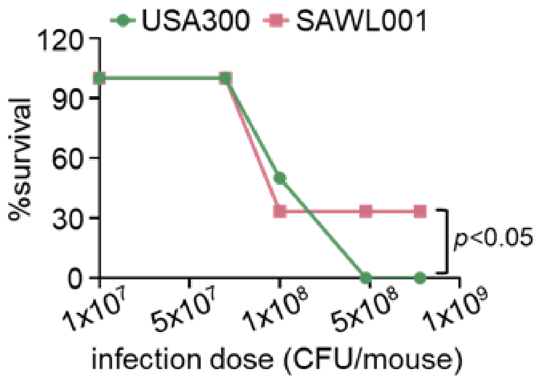

Strain	LD_{50}
USA300	1.0 × 10^8 CFU/mouse
SAWL001	8.4 × 10^7CFU/mouse

Fig. S5. The lethality of USA300 and SAWL001 in mouse sepsis model. Mice (n=6 per group) were infected intraperitoneally with the indicated bacterial doses. The graph displays the percentage of surviving mice at 48 h post-infection. The table below lists the LD_{50} values for USA300 and SAWL001 based on the survival data.

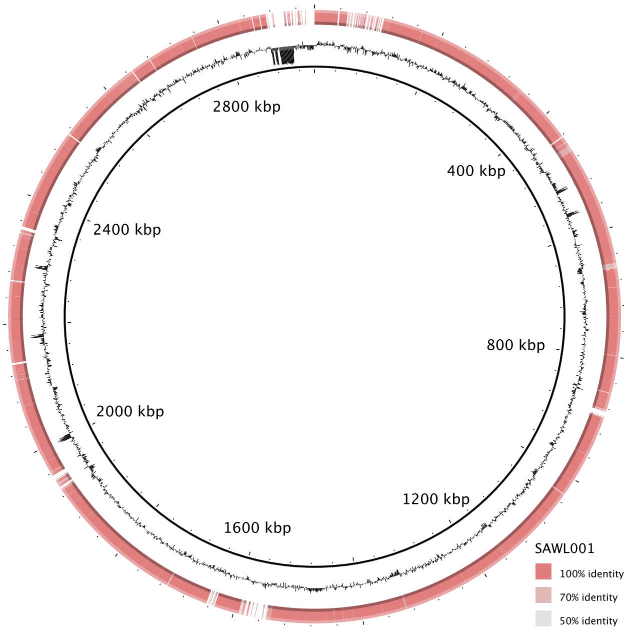

Fig. S6. Comparative circular genome map of the SAWL001 and USA300 chromosome. The map illustrates the nucleotide similarity of SAWL001 compared to the USA300_FPR3757 reference genome. From the outermost circle moving inwards: percent nucleotide similarity, GC skew plot, and the position of USA300 chromosome. GC skew plot was calculated with a 1,000 bp-window size. The innermost circle shows the coordinate scale of the USA300 chromosome with major and minor ticks at 200- and 50-kilobase intervals, respectively.

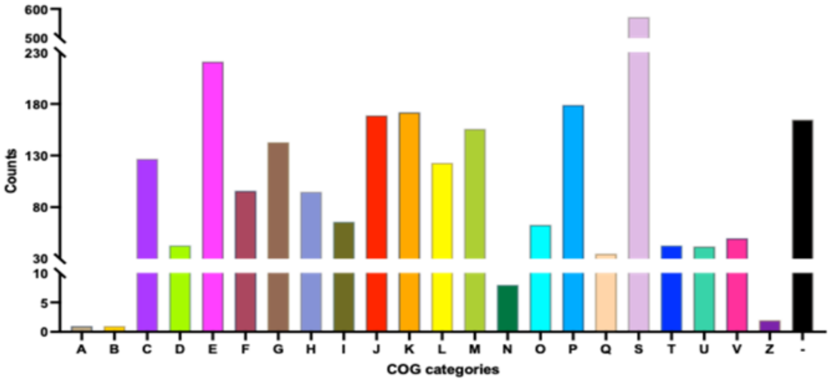

A: RNA processing and modification
B: Chromatin structure and dynamics
C: Energy production and conversion
D: Cell cycle control, cell division, chromosome partitioning
E: Amino acid transport and metabolism
F: Nucleotide transport and metabolism
G: Carbohydrate transport and metabolism
H: Coenzyme transport and metabolism
I: Lipid transport and metabolism
J: Translation, ribosomal structure and biogenesis
K: Transcription
L: Replication, recombination and repair
M: Cell wall/membrane/envelope biogenesis
N: Cell motility
O: Posttranslational modification, protein turnover, chaperones
P: Inorganic ion transport and metabolism
Q: Secondary metabolites biosynthesis, transport and catabolism
S: Unknown
T: Signal transduction mechanisms
U: Intracellular trafficking, secretion, and vesicular transport
V: Defense mechanisms
Z: Cytoskeleton
-: Unassigned

Fig. S7. Statistics of clusters of orthologous groups (COGs) of SAWL001. The proteins of SAWL001 were assigned to functional categories using the COG database (4). The bar chart illustrates the total number of proteins distributed across the major COG categories. Each category is represented by a single-letter code, with the full description provided in the accompanying legend.

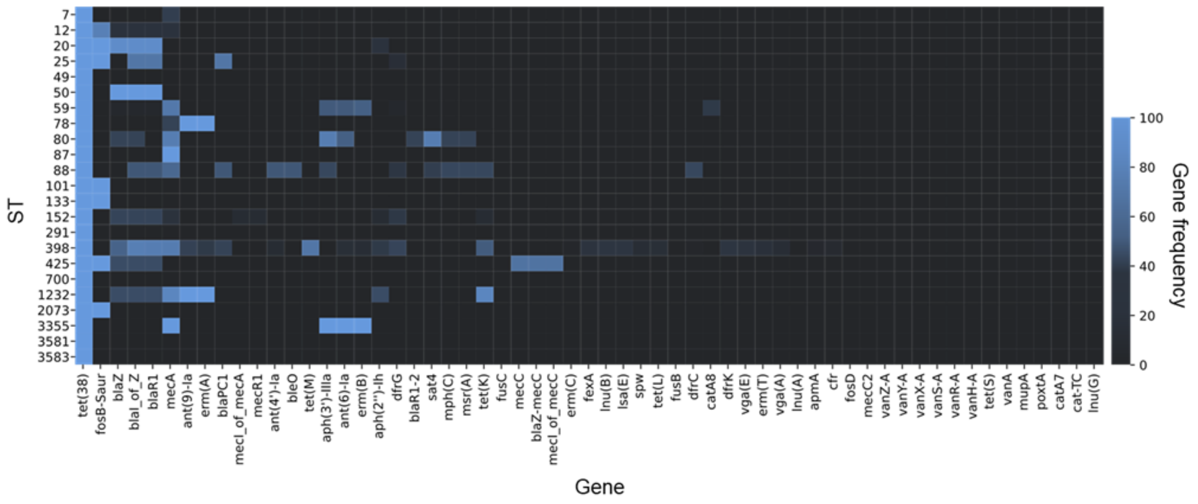

Fig. S8. Filtered sequence types from Figure 4a. The analysis presented in Figure 4a includes only sequence types (STs) that have been assigned to a known clonal complex (CC). The rows, each representing a distinct ST, are arranged in ascending numerical order. The columns correspond to individual genes, which are presented in the same order as in Figure 4a.

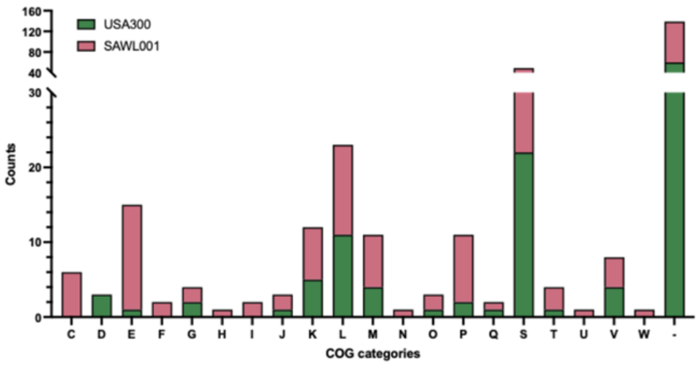

C: Energy production and conversion
D: Cell cycle control, cell division, chromosome partitioning
E: Amino acid transport and metabolism
F: Nucleotide transport and metabolism
G: Carbohydrate transport and metabolism
H: Coenzyme transport and metabolism
I: Lipid transport and metabolism
J: Translation, ribosomal structure and biogenesis
K: Transcription
L: Replication, recombination and repair
M: Cell wall/membrane/envelope biogenesis
N: Cell motility
O: Posttranslational modification, protein turnover, chaperones
P: Inorganic ion transport and metabolism
Q: Secondary metabolites biosynthesis, transport and catabolism
S: Unknown
T: Signal transduction mechanisms
U: Intracellular trafficking, secretion, and vesicular transport
V: Defense mechanisms
W: Extracellular structures
-: Unassigned

Fig. S9. Statistics of differentially expressed (DE) genes in comparison of SAWL001 to USA300. Genes identified as differentially expressed from a comparative transcriptomic analysis were functionally classified into COG categories. The bar chart displays the number of DEGs within each major functional category, distinguished by their direction of regulation. Pink bars indicate the number of genes upregulated in SAWL001 relative to USA300, while green bars indicate the number of genes downregulated in SAWL001, in other words, upregulated in USA300.

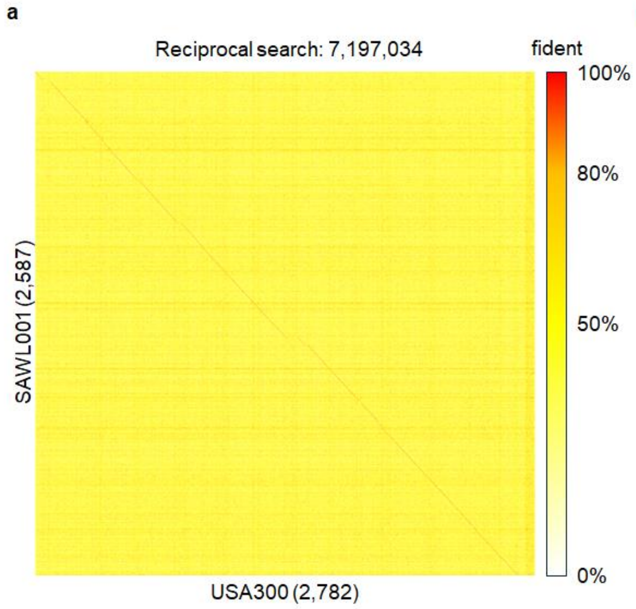

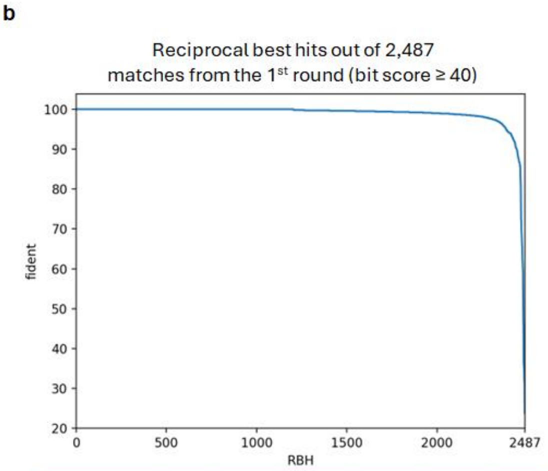

fident ≥	0.95	0.9	0.8	0.7	0.6	0.5
count	2383	2443	2465	2468	2476	2478
proportion	95.82	98.23	99.12	99.24	99.56	99.64

Fig. S10. Fractional identities of each gene pairs during the reciprocal best hit searching. (a) To identify reciprocal best hits (RBHs) between the SAWL001 and USA300 proteomes, an exhaustive all-vs-all sequence comparison was performed, encompassing a total of 7,197,034 pairwise alignments. (b) The maximum curvature point was identified by calculating the first and second derivatives of the fractional identity curve and used as a statistically robust threshold for defining orthologous pairs.

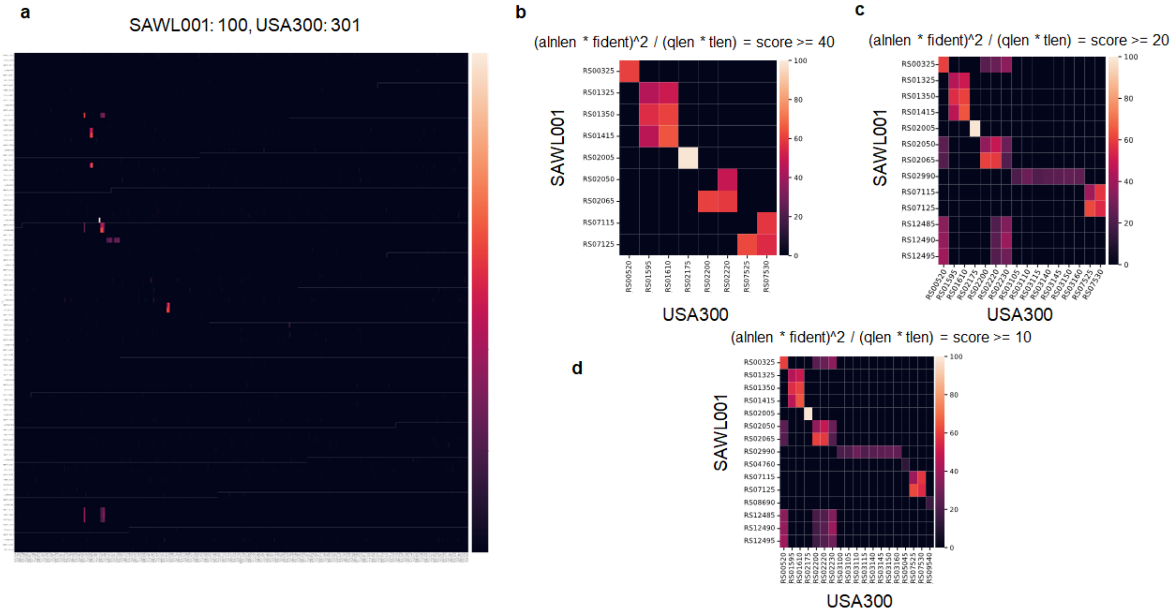

Fig. S11. Reciprocal best hit (RBH) analysis between SAWL001 and USA300 proteomes under different cutoff thresholds. (a) Heatmap of all-vs-all sequence comparisons between 100 proteins from SAWL001 and 301 proteins from USA300. Each cell represents the similarity score of a gene pair, with color intensity indicating fractional identity.

(b–d) Identification of orthologous pairs under varying thresholds of the similarity score, calculated as $(\text{alnlen} \times \text{fident})^2 / (\text{qlen} \times \text{tlen})$. (b) A stringent cutoff (≥40) highlights only high confidence orthologs. (c) A moderate cutoff (≥20) detects additional homologous candidates. (d) A relaxed cutoff (≥10) captures further potential matches but also introduces background noise. Together, these panels illustrate how threshold stringency strongly influences the resolution and reliability of ortholog assignments.

Table S1. Bacterial strains used in this study

Strains	Relevant features	Source or reference
S. aureus SAWL001	Korean clinically isolate	5
S. aureus USA300	USA300 TCH1516 derivative by curing the plasmid pUSA300HOUMR	6, 7
S. aureus HG003	wild type	6
S. aureus Newman	wild type	7
S. aureus RN4220	wild type	6
S. aureus MW2	wild type	6
E. coli DH5α	wild type	6
E. coli DH5α pFD116-*blaR1*	*E. coli* DH5α with plasmid pFD116-*blaR1*	This study
S. aureus RN4220 pFD116-*blaR1*	RN4220 with plasmid pFD116-*blaR1*	This study
S. aureus SAWL001 pFD116-*blaR1*	SAWL001 with plasmid pFD116-*blaR1*	This study

Table S2. List of primers used for cloning and analysis of gene expression

primers	Sequence	Reference
*mecA*_Fw	5’-GAATGCAGAAAGACCAAAGC-3’	8
*mecA*_Rv	5’-TTCTTTGGAACGATGCCTAT-3’	8
*purK*_Fw	5’-GGTGGTGGTCAGCTTGGAAA-3’	This study
*purK*_Rv	5’-TTGAGTGCCTTTTCATCGTCA-3’	This study
*gapA*_Fw	5’-TGCAAGGTCGTTTCACAGGT-3’	This study
*gapA*_Rv	5’-GGGATGATGTTTTCTGCCGC-3’	This study
*blaR1*_sgRNA_Fw	5’- CTAAGAGAATTTAATAG-GGATGAA-3’	This study
*blaR1*_sgRNA_Rv	5’- AAACTTCATCCCTATTAAATTCTC-3’	This study
pFD116_sgRNA_Rv	5’-AATGACTAGAGAAGAAGACT-3’	This study
*blaR1*_Fw	5’-AGCCCTTACAAAGCGATTACCA-3’	This study
*blaR1*_Rv	5’-TGACGGTCAAGTCC-AAACAGT-3’	This study
blaI_Fw	5’-TGAAATATCTATGGCTGAATGG-3’	This study
blaI_Rv	5’-ATCGTCTTATCGCTAACTTC-3’	This study

Reference

1. Nguyen LT, Schmidt HA, von Haeseler A, Minh BQ. IQ-TREE: a fast and effective stochastic algorithm for estimating maximum-likelihood phylogenies. Mol Biol Evol. 2015 Jan;32(1):268-74. doi: 10.1093/molbev/msu300.

2. Harris SR, Feil EJ, Holden MT, Quail MA, Nickerson EK, Chantratita N, Gardete S, Tavares A, Day N, Lindsay JA, Edgeworth JD, de Lencastre H, Parkhill J, Peacock SJ, Bentley SD. Evolution of MRSA during hospital transmission and intercontinental spread. Science. 2010 Jan 22;327(5964):469-74. doi: 10.1126/science.1182395.

3. Ahrenfeldt J, Skaarup C, Hasman H, Pedersen AG, Aarestrup FM, Lund O. Bacterial whole genome-based phylogeny: construction of a new benchmarking dataset and assessment of some existing methods. BMC Genomics. 2017 Jan 5;18(1):19. doi: 10.1186/s12864-016-3407-6.

4. Bosi E, Monk JM, Aziz RK, Fondi M, Nizet V, Palsson BØ. 2016. Comparative genome-scale modelling of *Staphylococcus aureus* strains identifies strain-specific metabolic capabilities linked to pathogenicity. Proceedings of the National Academy of Sciences 113:E3801-E3809.

5. Kang GS, Jung YH, Kim HS, Lee YS, Park C, Lee KJ, Cha JO. 2016. Prevalence of Major Methicillin-Resistant *Staphylococcus aureus* Clones in Korea Between 2001 and 2008. Annals of Laboratory Medicine 36:536-541.

6. Kim JH, Lee Y, Kim I, Chang J, Hong S, Lee NK, Shum D, Baek S, Kim W, Jang S, Lee W. 2024. Reducing Peptidoglycan Crosslinking by Chemical Modulator Reverts β-lactam Resistance in Methicillin-Resistant *Staphylococcus aureus*. Adv Sci (Weinh) 11:e2400858.

7. Chang J, Lee C, Kim I, Kim J, Kim JH, Yun T, Hooper DC, Walker S, Lee W. 2025. Environmental cues in different host niches shape the survival fitness of *Staphylococcus aureus*. Nature Communications 16:6928.

8. Kim J-U, Cha C-H, An H-K, Lee H-J, Kim M-N. 2013. Multiplex Real-Time PCR Assay for Detection of Methicillin-Resistant *Staphylococcus aureus* (MRSA) Strains Suitable in Regions of High MRSA Endemicity. Journal of Clinical Microbiology 51:1008-1013.
